# REGSTATTOOLS: freeware statistical tools for the analysis of disease population databases used in health and social studies

**DOI:** 10.1186/1471-2458-13-201

**Published:** 2013-03-07

**Authors:** Laura Esteban, Ramon Clèries, Jordi Gálvez, Laura Pareja, Josep Maria Escribà, Xavier Sanz, Ángel Izquierdo, Jaume Galcerán, Josepa Ribes

**Affiliations:** 1 Cancer Registry of Catalonia, Plan for Oncology of the Catalan Government, IDIBELL, Hospital Duran i Reynals. Av. Gran Via de l’Hospitalet, 199-203, 08908 – L’Hospitalet de Llobregat, Catalonia, Spain; 2 Department of Clinical Sciences, University of Barcelona, Barcelona 08907, Spain; 3 Epidemiology Unit and Cancer Registry of Girona, Institut Català d’Oncologia (ICO), Girona 17004, Catalonia, Spain; 4 Tarragona Cancer Registry. Foundation Society for Cancer research and Prevention, Pere Virgili Health Research Institute, Reus 43201, Spain

**Keywords:** Web-application, Prediction, Standardized incidence mortality ratio, Annual percent change, Net percent change of rates, Relative survival

## Abstract

**Background:**

The repertoire of statistical methods dealing with the descriptive analysis of the burden of a disease has been expanded and implemented in statistical software packages during the last years. The purpose of this paper is to present a web-based tool, *REGSTATTOOLS*http://regstattools.net intended to provide analysis for the burden of cancer, or other group of disease registry data. Three software applications are included in *REGSTATTOOLS*: *SART* (analysis of disease’s rates and its time trends), *RiskDiff* (analysis of percent changes in the rates due to demographic factors and risk of developing or dying from a disease) and *WAERS* (relative survival analysis).

**Results:**

We show a real-data application through the assessment of the burden of tobacco-related cancer incidence in two Spanish regions in the period 1995–2004. Making use of *SART* we show that lung cancer is the most common cancer among those cancers, with rising trends in incidence among women. We compared 2000–2004 data with that of 1995–1999 to assess percent changes in the number of cases as well as relative survival using *RiskDiff* and *WAERS*, respectively. We show that the net change increase in lung cancer cases among women was mainly attributable to an increased risk of developing lung cancer, whereas in men it is attributable to the increase in population size. Among men, lung cancer relative survival was higher in 2000–2004 than in 1995–1999, whereas it was similar among women when these time periods were compared.

**Conclusions:**

Unlike other similar applications, *REGSTATTOOLS* does not require local software installation and it is simple to use, fast and easy to interpret. It is a set of web-based statistical tools intended for automated calculation of population indicators that any professional in health or social sciences may require.

## Background

An aim of public health assessment involves describing the health status of a defined population by looking at their changes over time or by comparing their health events to events occurring in other populations. Descriptive epidemiology of cancer, for example, may assess the size of the problem that cancer poses to health, measuring the risk in the same population at different periods of time [1]. To account for rising trends of cancer in a population or to compare populations of different sizes, rates are usually developed to provide the number of events per population unit [2], whereas the number of cancer cases is used to measure the burden of cancer into the health system [3].

Worldwide, statistical methods for descriptive analysis has been expanded and implemented in statistical software packages during the last years. The most comprehensive coverage of statistical methods for analyzing cancer data is SEER*Stat [4], whereas user-friendly statistical software packages for specific time-trend modelling of rates have also been developed to measure the burden of cancer and its projections [5]–[7] changes in trends [8], and survival analysis [9]–[11].

The purpose of this paper is to present a set of web-based tools, *REGSTATTOOLS*http://regstattools.net, in order to provide a very easy-intuitive way to carry out statistical analyses. The user must upload the predefined file to *REGSTATTOOLS* web-page to obtain for a determined disease and a period of time: (i) descriptive statistics, (ii) the estimated annual percent change in rates; (iii) the standardized incidence or mortality ratio comparing two time periods or two geographical areas; (iv) the prediction of the expected incident or death cases; (v) the assessment of the differences for incidence or deceased cases between two different time points or two geographical areas in order to clarify the role of the changes on demographic factors and the risk of developing or dying from the disease, and finally, (vi) comparing observed and relative survival.

In this paper *REGSTATTOOLS* is introduced describing its use through an example on the assessment of the burden of tobacco-related cancer incidence in two Spanish regions during the period 1995–2004.

## Implementation

### Descriptive statistics for rates

Suppose that we want to assess the burden of a disease in a certain population of size N during a certain period of time. Consider that we have observed X cases of the disease under study, therefore the crude rate (CR) is defined as X/N. The CR is the simplest and most straightforward summary measure of the population’s diseases under study. But the events may be strongly related to age, so the age-specific events will differ greatly from one another, therefore it is of interest to calculate the age-specific rates. The use of a world standard population [12] and direct standardization (or any other adjustment procedures) seek to provide numbers and comparisons that minimize the influence of age and/or other extraneous factors through the age-standardized rates (ASR) [13]–[15]. These ASRs can be also truncated for the age groups of interest. In cancer, the calculation of truncated rates (TR) over the age-range 35-64 [14] has been proposed, mainly because of doubts about the accuracy of age-specific rates in the elderly when diagnosis and recording of cancer may be much less certain. Finally, another useful summary measure of disease frequency is the cumulative rate [14,15] (CumR), which is the sum of the age-specific incidence rates, taken from birth to age 74, in a certain time period. CumR is an estimate of the cumulative risk (Cumulative Risk= 1-exp[−CumR]), which is the risk which an individual would have of developing an event of interest during a certain age-span if no other causes of death were in operation [14].

### Estimating the annual percent change in rates (EAPC)

In descriptive epidemiology, the evolution of incidence or mortality rates of certain disease during a determined time period can generate etiological hypotheses. The estimated annual percent change (EAPC) is one way to characterize trends in disease rates over time. This means that the rates are assumed to change at a constant percentage of the rate of the previous year [15]. Let us assume that we want to assess the EAPC of ASRs during a period of time (in cancer, usually years). Let *ASR*_*T*_ be the *ASR* for the T^*th*^ year, *T*; the time trend of the ASRs can be modelled through a Gaussian log-linear model,

(1) $$\log\left(\mathrm{AS}R_{T}\right)=\alpha +\mathrm{\beta \cdot T}$$

where *EAPC* = (*e*^*β*^ − 1)*·*100. The 95% confidence intervals of the EAPC can be easily derived through the standard errors of model (1) [15].

### Predicting the Expected number of incident (or death) disease cases by age group using the time trends of rates

Prediction of future disease burden is essential for effective health service planning, as it may be utilized by public health authorities to formulate prevention, diagnosis and treatment strategies [16]. Simple log-linear models can be used to make these predictions [17]. If we assume that the time trend of

$$\frac{C_{\mathrm{iT}}}{Y_{\mathrm{iT}}}$$,

where *C*_*iT*_ is the number of cases for the *i*^*th*^ age-group and period *T* and *Y*_*iT*_ are the corresponding person-years at risk is linear in its log-scale, the following log-linear model can be fitted to these rates:

(2) $$\text{log}\text{log}\left(\frac{C_{\mathrm{iT}}}{Y_{\mathrm{iT}}}\right)=\alpha_{i}+\beta_{i}\cdot \left(T-T_{0}\right)$$

where *T*_0_ is the reference time, *α*_*i*_ is the log-rate at *T*_*0*_ for the *i*^*th*^ age-group and *β*_*i*_ is the age-specific slope. A parsimonious version of this model can be also used assuming a common slope for each age group,

(3) $$\text{log}\left(\frac{C_{\mathrm{iT}}}{Y_{\mathrm{iT}}}\right)=\alpha_{i}+\beta \cdot \left(T-T_{0}\right)$$

where this model is known as the age-drift model. For these models we assume *C*_*iT*_ to follow the Poisson distribution [17]. However, the negative binomial distribution has been also used as an alternative to Poisson when there is evidence of “overdispersion” (higher variance than expected) in the data [18].

Prediction of incidence at a future time *F* can be made using the fitted model (2) or (3), and replacing *T* by *F* and *Y*_*iT*_ by *Y*_*iF*_ into the fitted model. Poisson and Negative Binomial distribution are both assumed for each model in (2) and (3). Therefore 4 models are assessed for the selection of the best fitting model to data. The assessment is made through the Akaike’s Information Criterion (AIC) [19] and the Chi-square test [17].

### Comparing risk between two groups (time periods or geographical areas): standardized incidence or mortality ratio (SIMR)

*SIMR* is used to determine if the occurrence of a disease in a target population is higher or lower than that occurrence in a reference one. For example, we can either compare the incidence of cancer in the same area in two different time periods or two different areas in the same time period. The *SIMR* can be calculated as

(4) $$\mathrm{SIMR}=\frac{D}{E}$$

where *D* is the number of observed events in the target population and *E* the number of expected events in this population using the incidence (or mortality) rates of the reference population [15].

### Assessment of differences due to risk and demographic factors when comparing disease rates of two populations

To assess differences for incidence or deceased cases between two different time points or two areas in order to clarify the role of the changes in demographic factors and the risk of developing or dying from a disease, we used the method of Bashir and Estève [20]. For example, we can compare the observed age-specific incidence cases of certain cancers in the period 1995–1999, $C_{i}^{1995-1999}$ , with the observed age-specific incidence cases in the period 2000–2004, $C_{i}^{2000-2004}$. Assuming eighteen 5-year age-groups, the observed percent net change of the difference in the total number of cases between both periods can be calculated as

(5) $$\mathrm{Net}\left(\%\right)=\frac{\sum_{i=1}^{18}\left(C_{i}^{2000-2004}-C_{i}^{1995-1999}\right)}{\sum_{i}^{18}C_{i}^{1995-1999}}$$

*Net(%)*, can be separated into two components: i) changes in size and age distribution (structure) of the population and ii) changes in the risk of developing the disease,

(6) $$\begin{array}{l} \text{Net}\;\left(\%\right)=\text{Risk}\;\left(\%\right)+\text{Population}\;\left(\%\right)\\ \;=\text{Risk}\;\left(\%\right)+\text{Size}\;\left(\%\right)\\ \;+\text{Structure}\;\left(\%\right) \end{array}$$

We note that in each age group we must take into account that rates into the period 2000–2004 must be considered as constant as well as rates into the period 1995–1999. If the population size is expected to increase by 10%, incident cases will also increase by 10%. The effect of population structure is estimated by comparing the rate observed in 1995–1999 and the rate expected in the 2000–2004, through applying the age specific rates observed in 1995–1999 to the population pyramid in 2000–2004. Lastly, the percent change not explained by percent change in the population will be considered to be due to the variation in risk of developing the disease [20]. We note that the net change can be also calculated for the CR [20]. Mathematical details of equation (6) can be found in the Additional file 1.

### Assessing survival of a cohort of patients diagnosed with a certain disease

The observed survival (OS) rate is the basic measure of the survival experience of a group of patients from the date of diagnosis to a certain time. However, information on the cancer patients’ causes of death might not be always suitable or it might be vague or unavailable [15,21]. Since the interest lies in describing mortality attributable to the disease under study, one method of estimating net survival, where the disease of interest is assumed to be the only possible cause of death [21], is relative survival (RS). It is interpreted as the probability to survive after diagnosis of the disease of interest. For a cohort of patients diagnosed with a certain disease, say cancer for example, the cumulative RS at time *T* is defined as

(7) $$\mathrm{RS}\left(T\right)=\frac{S_{O}\left(T\right)}{S_{E}\left(T\right)}$$

where *S*_*o*_(*T*) is the observed survival rate in the cohort of study and *S*_*E*_(*T*) is the expected survival of that cohort, this last estimated from a comparable general population life tables stratified by age, sex and calendar time and assume that the cancer deaths are a negligible proportion of all deaths [21]. The *RS*(*T*) can be calculated through estimating *S*_*o*_(*T*) by the Kaplan–Meier method and *S*_*E*_(*T*) using Hakulinen method [22]. The 95% confidence intervals of the *RS*(*T*) can be estimated through the standard errors of the log-transformation of *S*_*o*_(*T*) assuming *S*_*E*_(*T*) as a constant value [23]. Some interpretations about RS are not straightforward. Note that improvements in general mortality of the reference population affect *S*_*E*_(*T*) in Equation (7) [24]. Let’s suppose we want to compare 5-year RS of lung cancer between periods 1999–1994 (RS(5)=10,5%) and 1995–1999 (RS(5)=8,5%) among men in Catalonia (Spain) [25]. Although cancer mortality decreased in 2000–2004 compared to 1995–1999 in Catalonia [26], we observed a decrease of 5-year RS of lung cancer. It may suggest that RS(5) was worsening in 1995–1999 but the explanation is that *S*_*E*_(5) between both periods increased but *S*_*o*_(5) remained stable, and therefore RS(5) decreased [26]. In this line, two period comparison of RS for cancers with poor survival should be interpreted with caution [24].

### The set of web-based applications included in REGSTATTOOLS

*REGSTATTOOLS* (http://regstattools.net/) includes a set of web-based statistical applications running under Linux operating system installed in a web-server: *SART* (Statistical Analysis of Rates and Trends) [27], *RiskDiff* (a web tool for the analysis of the difference due to risk and demographic factors for incidence or mortality data) [28] and *WAERS* (Web-Assisted Estimation of Relative Survival) [29]. The web pages of all these applications were implemented using the server-side scripting language *PHP* and *HTML [*[30] whereas statistical computation has been implemented using *R* statistical software [31].

**Figure 1:**
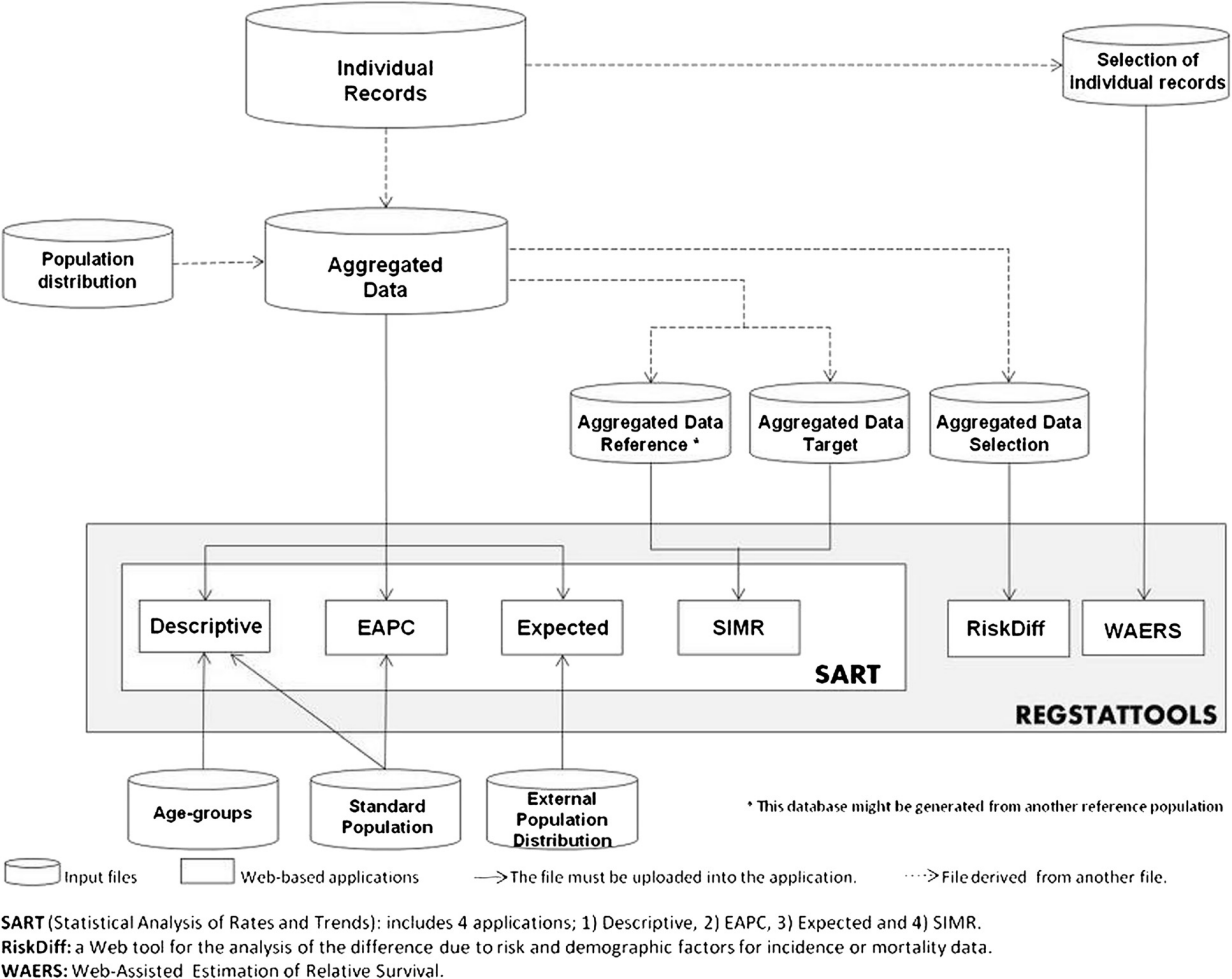
Schematic overview of Web-based applications included in REGSTATTOOLS and the required input files to perform each one of the statistical analyses.

Figure 1 shows an overview of the *REGSTATTOOLS* applications. Each application requires at least one input file to perform the corresponding statistical analysis. The user must have an *Individual Records* data file available which is a basis file to perform all the statistical analysis, since most of the data files used in the web applications are derived from this one. This basis file must contain the following patients’ variables: patient identification, sex, type of disease, age-month-year at diagnosis, status of the patient (dead or alive), month-year of the death, and finally, the follow-up time in years. The *SART* applications require a total of 6 files to be uploaded whereas *RiskDiff* and *WAERS* applications only require one file each.

The *SART* applications [27] require an *Aggregated Data* file that must contain the following columns: sex, age-group, incidence or mortality year, type of disease, cases and person-years at risk. To perform a descriptive analysis of the disease rates, the user can make use of the *Descriptive* application after preparing an age-groups file and a standard population’s file. The time trends analysis of rates can be performed using the *EAPC* application which also requires a standard population file. The application *Expected* allows a prediction of the expected number of cases in a future period or in other geographical areas, using the aggregated data and a file which contains an external population distribution (person-years at risk by age-group). The comparison of risk between two groups can be performed through *SIMR* application which requires a partition of the *Aggregated Data* file into two files, each one with data of the corresponding time period. Another possibility could be comparing these data with data from another area in the same time period; therefore, two files are required. In this line, note that the user must prepare 6 files to fully run *SART*.

The *RiskDiff* application [28] has been developed to perform the analysis described in section *Assessment of differences due to risk and demographic factors when comparing disease rates of two populations*. It requires information on the number of cases and person years at risk by age-group in each one of the two periods or two geographical areas to be compared. In this line, the *Aggregated Data Selection* file must contain 2 columns for each period/area compared: one column referring to person-years and another column referring to number of cases.

Finally, the RS must be obtained through the *WAERS* application [29] which requires a *Selection of Individual Records* file with the following variables: patients ID, age and year at beginning of study, sex, years of follow-up and vital status (death or alive).

We will refer to AF throughout the paper where additional figures and tables can be found, and those that are related to the example section.

## Results

To illustrate the use of *REGSTATTOOLS* we will assess the burden of tobacco-related cancer in Girona and Tarragona provinces (northeast of Spain), which cover 1,200,000 inhabitants in 2001. Incidence data were provided in the *Individual Records* data file which includes data from all patients diagnosed with larynx, oral cavity, pharynx, oesophagus, stomach, lung, pancreas and kidney tumours in the period 1995–2004. Patients were followed-up until December 31st 2007. A total 10297 men and 2695 women (mean age 66.1 and 70.1 years, respectively) were included in the analysis. This file is the basis to perform the statistical analyses (Table 1) from which we can create the data file to be uploaded into *SART* by means of aggregated data in eighteen 5-year age-groups (or other age groups according to user’s requirements).

**Table 1:** File example of individual records

**Patient_ID**	**Sex**	**d_group**	**d_age**	**i_month**	**i_year**	**f_month**	**f_year**	**Status**	**Follow_up**
1	1	Lung	84	3	1995	3	1995	1	0.08
2	1	Lung	65	2	1995	12	1999	1	0.83
3	1	Lung	63	3	1995	5	1999	1	4.17
…	…	…	…	…	…	…	…	…	…
194	2	Lung	72	2	1995	8	1995	1	0.50
195	2	Lung	42	9	1995	11	1995	1	0.17
196	1	Lung	72	10	1995	1	1996	1	0.25
197	1	Lung	52	5	1995	12	2008	0	12.58
198	1	Lung	79	1	1996	9	1997	1	1.67
…	…	…	…	…	…	…	…	…	…
6087	1	Larynx	59	6	2000	8	2004	1	4.17
6088	2	Larynx	68	7	2001	8	2004	1	3.08
6089	2	Larynx	53	3	2000	12	2006	0	6.75
6090	1	Larynx	87	7	1995	12	2006	0	11.42
…	…	…	…	…	…	…	…	…	…
12989	1	Kidney	50	7	2002	5	2003	1	0.83
12990	2	Kidney	90	7	2003	7	2003	1	0.08
12991	1	Kidney	49	6	2004	6	2004	1	0.08
12992	1	Kidney	67	4	2004	6	2004	1	0.08

**Table 2:** Girona and Tarragona aggregated data

**Sex**	**Age.group**	**Year**	**Group**	**Cases**	**Population**
1	1	2000	Kidney	0	27251
1	2	2000	Kidney	0	27741
1	3	2000	Kidney	0	29381
…	…	…	…	…	…
2	16	2004	Larynx	0	26115
2	17	2004	Larynx	0	19665
2	18	2004	Larynx	0	15737
…	…	…	…	…	…
1	1	2000	Lung	0	27251
1	2	2000	Lung	0	29381
…	…	…	…	…	…
2	16	2004	Stomach	8	26115
2	17	2004	Stomach	19	19665
2	18	2004	Stomach	15	15737

**Table 3:** ***WAERS *output for lung cancer incidence in Girona and Tarragona. 4 executions by period and sex**

**Men**											
**1995-1999**						**2000-2004**					
Risk	T	RS	LCI	UCI	OS	Risk	T	RS	LCI	UCI	OS
2252	0	0.999	0.998	1	0.999	2514	1	0.325	0.307	0.344	0.313
2250	1	0.292	0.273	0.312	0.282	775	2	0.191	0.175	0.207	0.179
616	2	0.156	0.141	0.173	0.146	436	3	0.15	0.135	0.165	0.136
319	3	0.113	0.1	0.128	0.103	299	4	0.129	0.116	0.144	0.114
224	4	0.096	0.084	0.11	0.085	219	5	0.115	0.101	0.13	0.099
186	5	0.083	0.071	0.096	0.071	140	6	0.106	0.093	0.121	0.089
155	6	0.074	0.063	0.087	0.062	89	7	0.101	0.087	0.117	0.082
135	7	0.066	0.055	0.079	0.054	40	8	0.089	0.073	0.108	0.07
117	8	0.056	0.046	0.068	0.044	17	9	0.065	0.045	0.093	0.049
89	9	0.051	0.042	0.063	0.039						
61	10	0.047	0.037	0.059	0.035						
41	11	0.04	0.031	0.052	0.029						
21	12	0.037	0.028	0.05	0.026						
12	13	0.036	0.025	0.05	0.024						
4	14	0.027	0.014	0.053	0.018						
**Women**											
**1995-1999**						**2000-2004**					
Risk	T	RS	LCI	UCI	OS	Risk	T	RS	LCI	UCI	OS
240	1	0.275	0.222	0.339	0.267	312	1	0.339	0.289	0.397	0.33
61	2	0.174	0.131	0.232	0.166	100	2	0.227	0.184	0.281	0.218
38	3	0.135	0.097	0.189	0.127	65	3	0.174	0.135	0.224	0.164
29	4	0.123	0.086	0.177	0.114	42	4	0.147	0.11	0.196	0.137
26	5	0.103	0.071	0.157	0.096	31	5	0.14	0.104	0.189	0.128
22	6	0.093	0.061	0.142	0.083	20	6	0.128	0.092	0.179	0.115
19	8	0.085	0.054	0.033	0.074	9	7	0.073	0.037	0.143	0.064
15	9	0.08	0.05	0.128	0.069	1	9	0.086	0.044	0.169	0.064
12	10	0.08	0.05	0.129	0.069						
7	11	0.082	0.051	0.031	0.069						
3	12	0.083	0.052	0.134	0.069						
1	13	0.088	0.055	0.141	0.069						

The Descriptive application has been used after preparing an age-groups file (Additional file 1: Table Aff1) and a world standard population’s file (Additional file 1: Table Aff2). Since we are analysing rates, note that Girona and Tarragona population’s person-years at risk for each sex-age-group-year are required. We made use of the Catalan Institute of Statistics population’s distribution (available at: www.idescat.net). Merging a *Population-Distribution* (person-years) data file (Additional file 1: Table Aff3) and the previous one, we can obtain the *Aggregated Data* file (Table 2). Results of the descriptive analysis of tobacco-related cancer in Girona and Tarragona showed that ASR of lung cancer is the first ranking in men’s (ASR=48.27 per 100,000 men-years at risk) and the second one in women’s (ASR=5.12 per 100,000 women-years at risk) in the ranking of the tumours of interest (Additional file 1: Figure Aff1). Lung cancer has the highest risk among the tobacco-related cancers in men (CumR=6%, 6 per 100 men are at risk of developing lung cancer before 75 years old, Additional file 1: Table Aff4). In women, lung and stomach cancers have the highest risk among the tobacco-related cancers (CumR= 0.58%, less than 1 per 100 women are at risk of developing lung or stomach cancer before 75 years old, Additional file 1: Table Aff4).

**Figure 2:**
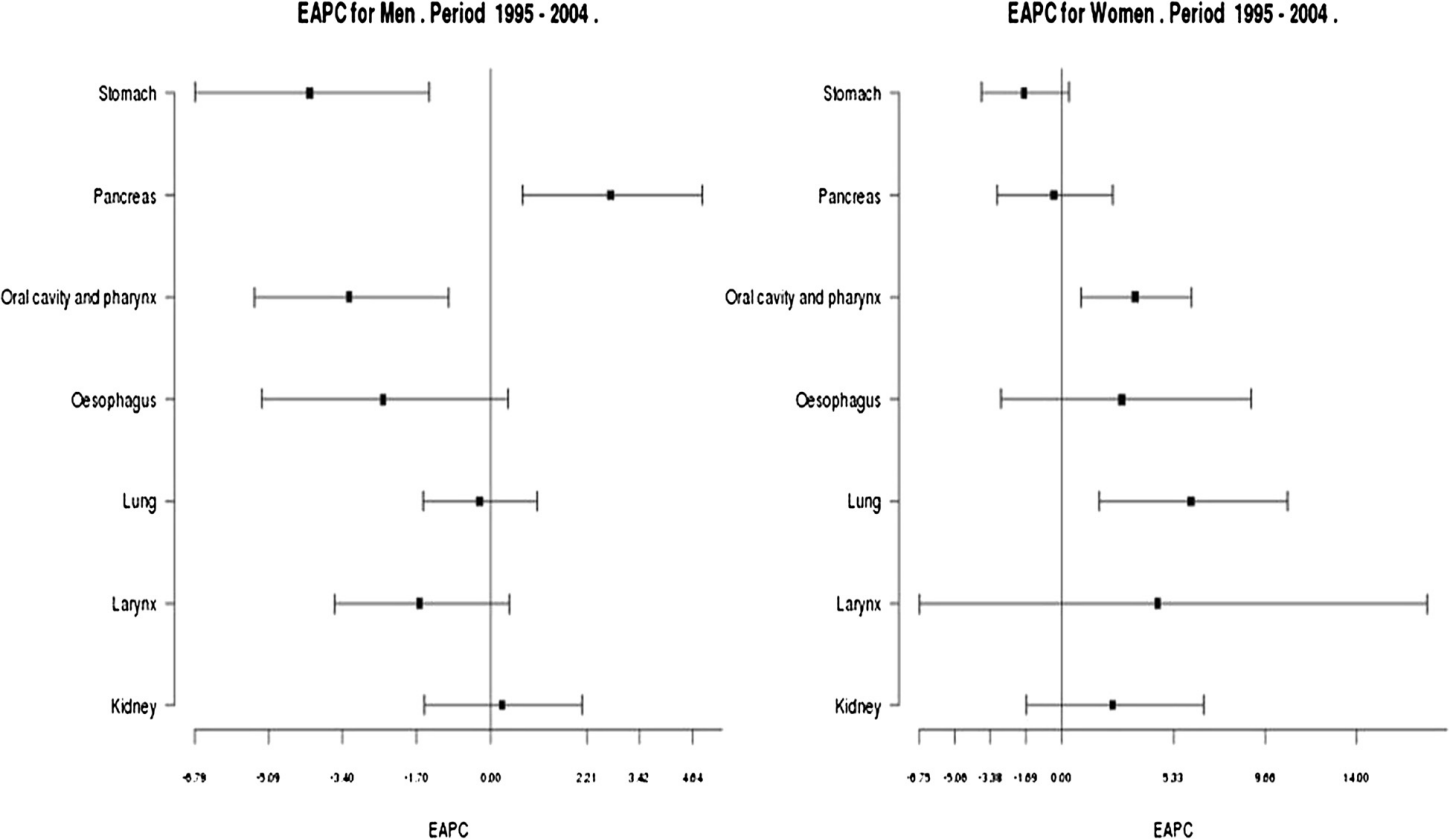
**Estimated annual percent change incidence cancer in Girona and Tarragona.** 1995–2004.

EAPC of tobacco-related cancer rates during the period 1995–2004 in both sexes is depicted in Figure 2. EAPC can be interpreted as the annual change of rates in the magnitude of a trend. If the confidence interval includes 0 value, the change is not statistically significant whereas if it is not included, it is interpreted as a significant increase (if it is positive) or decrease (if it is negative). In men, lung cancer remained stable (EAPC= −0.24, 95%CI: -1.54, 1.08) whereas some tumours showed a significant decreasing trend such as stomach (EAPC= −4.14, 95%CI: -6.79, -1.42) and oropharynx (EAPC= −3.23, 95%CI: -5.43, -0.97) and a significant increasing trend such as pancreas (EAPC= 2.77, 95%CI: 0.73, 4.85) (Additional file 1: Table Aff5). In women, significant increases in the study period were detected in lung (EAPC=6.16, 95%CI: 1.81, 10.70) and oropharynx tumours (EAPC=3.52, 95%CI: 0.91, 6.19), (Additional file 1: Table Aff5).

**Figure 3:**
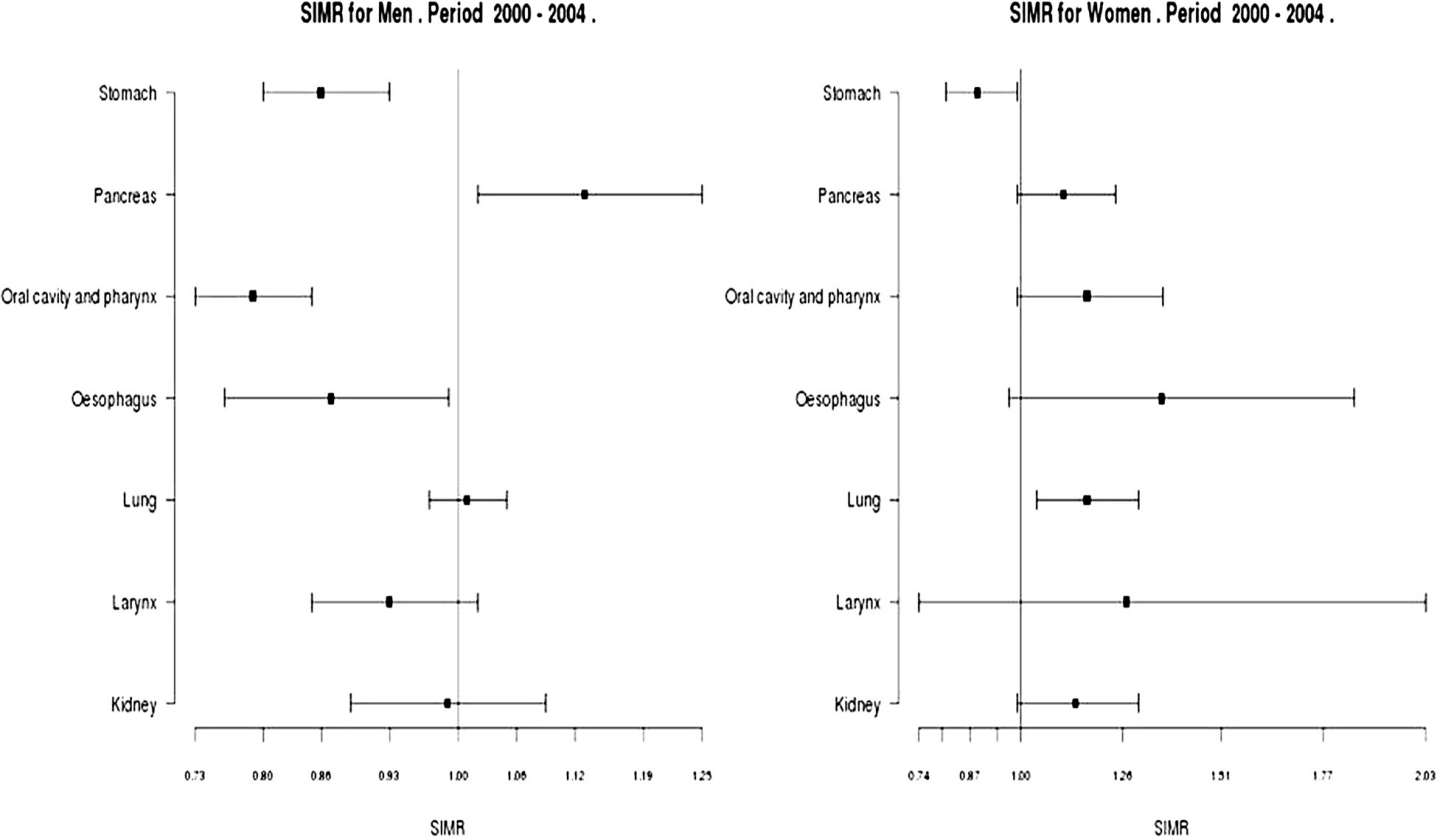
**Standardized Incidence Ratio incidence cancer in Girona and Tarragona.** 2000–2004 vs. 1995–1999.

Although some cancers did not show a significant time trend during the whole time period, there might be a change in the risk of developing the cancer at the individual level. Therefore, we could prepare two datasets, one which includes 1995–1999 aggregated data (reference period) and another with 2000–2004 aggregated data (target period) (Additional file 1: Table Aff6) and making use of the SIMR application we could assess the change in risk of developing lung cancer between these periods. SIMR can be interpreted as the increase or decrease of risk of developing or of dying from a disease comparing the observed number of cases of a population respect to a reference one. If the SIMR’s confidence interval includes the value 1, the SIMR is not statistically significant, otherwise, it is interpreted as a significant excess or reduced risk of having a disease or dying from a disease. Figure 3 compares the SIMRs for each cancer site analyzed between men and women. In women, a significant 17% higher lung cancer incidence is observed with 2000–2004 versus 1995–1999 (SIMR=1.17, 95% CI: 1.04, 1.30, Additional file 1: Table Aff7).

**Figure 4:**
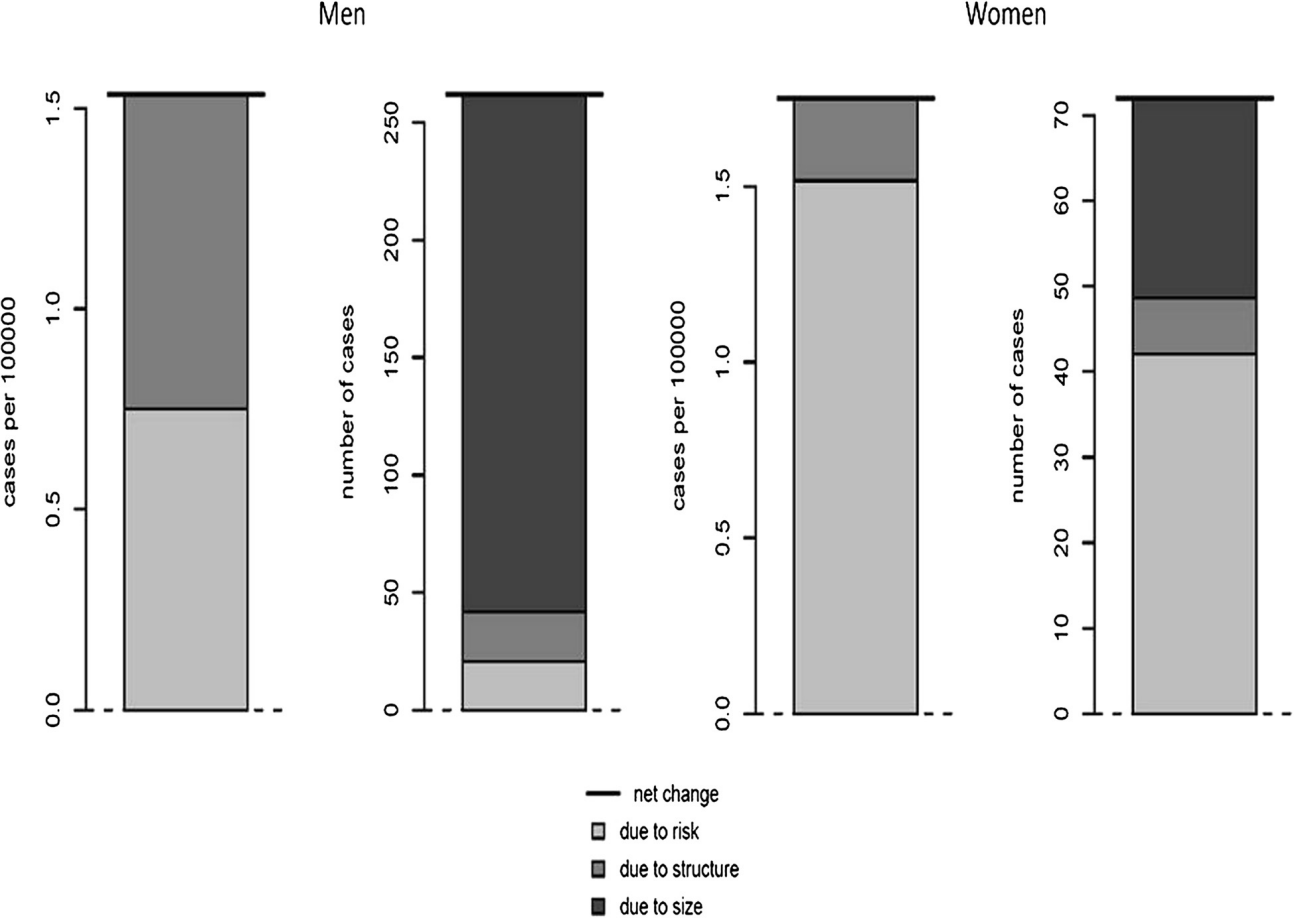
**Partition of the net change between risk, structure and size in lung cancer incidence from Tarragona and Girona.** 2000–2004 vs. 1995–1999: (I) Difference in the crude rate (number of lung cancer cases per 100,000 person-years) between 1995–1999 respect to 2000–2004 among men (**A.1**) and among women (**A.2**); (II) Difference in the absolute number of lung cancer cases between 1995–1999 respect to 2000–2004 among men (**B.1**) and among women (**B.2**). (Note that differences in the number of cases are partitioned into those due to risk and those due to population structure and population size.

To assess the changes in the number of incident lung cancer cases among time periods making use of *RiskDiff* we have prepared an *Aggregated Data Selection* file (Additional file 1: Table Aff8). Assuming that incidence rates in the period 2000–2004 are constant, as well as rates in the period 1995–1999 are also constant, we have compared cancer incidence in both periods. We found that the number of cases was 11.63% higher in 2000–2004 compared to 1995–1999 among men, whereas among women this change was about 30.0% (Additional file 1: Table Aff9). Figure 4 shows the contribution of demographic factors and the risk of developing lung cancer to this net change in the CR, cases per 100,000 person-years, and absolute number of cases. In men, changes in the CR due to risk and population structure are similar (Risk: 0.75 and Structure: 0.78 cases per 100,000 person-years) whereas changes in risk contribute the most among women (Risk: 1.52 and Structure: 0.24 cases per 100,000 person-years). In men net change in absolute number of cases was basically due to a change in population size (9.78%), while changes in population structure and changes in the risk of developing lung cancer were much less important, 0.95% and 0.91% respectively. On the contrary, in women, the net change in the absolute number of cases was mainly attributable to an increased risk of developing lung cancer (17.53%) while the changes in size and population structure play a less important role (9.76% and a 2.72%, respectively) (Additional file 1: Table Aff9 and Figure 4). These results are in agreement with those observed in the SIMR analyses.

We also assessed the evolution of the 5-year RS rates of lung cancer between the time period 2000–2004 and the time period 1995–1999 using *WAERS* through a *Selection of Individual Records* file (Additional file 1: Table Aff10). Table 3 shows the *WAERS* output, where we found that 5-year RS improved significantly among men (5-year RS 1995-1999=8.3%, 95% CI: 7.1%-9.6% versus 5-year RS 2000-2004=11.5%, 95% CI: 10.1%-13.0%) whereas these differences between RS were not statistically significant among women (5-year RS 1995-1999=10.6%, 95% CI: 7.1%-15.7% versus 5-year RS 2000-2004=14.0%, 95% CI: 10.4%-18.9%).

Finally, we predicted the burden of lung cancer for the year 2014 in Catalonia through the *Expected* application of *SART* making use of the predicted population for Catalonia in 2014 (Additional file 1: Table Aff11). The application selected the age-drift models (Additional file 1: Table Aff12) as the best fitting ones, predicting 777 cases among women with an ASR of 10.74 cases per 100,000 women-years and 3085 cases among men with an ASR of 46.82 cases per 100,000 men-years (Additional file 1: Table Aff13). We can also observe the cases by age-group in Additional file 1: Table Aff14 (the output of the application).

## Discussion

There are many “stand-alone” web pages which are designed to perform only a single statistical test or calculation. *REGSTATTOOLS* is a website which performs an entire suite of calculations for registry data, with a logical organization and consistent user interface. *REGSTATTOOLS* incorporates a flexible data import with a variety of methods in order to facilitate the widespread use of these applications with a basic statistical knowledge. The development of *REGSTATTOOLS*’ applications is an ongoing process which has been implemented with the current version of *SART*, *RiskDiff* and *WAERS*. In this paper, the use of *REGSTATTOOLS’* applications was illustrated by analyzing population-based cancer registry data. However, it can be used to analyze other disease registry data such as diabetes or any other chronic disease.

Up to date of publication *SART, WAERS* and *RiskDiff* were accessed 3,179 times in total. Although each application was available on the internet at a different period of time, each one shows different percentage of use by user type (see Additional file 1: Table Aff15). Cancer registries are the main users of *SART* (65.94%) and *WAERS* (60.71%) whereas they are the second in the ranking of *RiskDiff* (35.71%) users. Universities and research centres are also major *WAERS* (26.16%) and *RiskDiff (42.86%)* users.

*SART* includes the calculation of disease rates and other indicators in the same application with no requirement of software installation [27]. *WAERS* is a web-based survival-specific application to perform basic RS analysis [29]. Nowadays, mortality rates from all European countries, the United States of America, Canada and Argentina have been incorporated into *WAERS*. Any *WAERS* user from all these countries can estimate RS making use of these mortality tables. More advanced survival analysis can be carried out using other statistical software [9]–[11] that requires previous installation in the user’s local computer as well as some technical skills about multivariate analysis. On the other hand and to our knowledge, *RiskDiff* is the only available web tool that can perform a statistical analysis which identifies which percentage of change in disease rates between two time periods or areas are due to changes in demographic factors, and which are due to changes in risk [28].

Some limitations should be noted in these applications in their current versions. *SART* does not provide confidence intervals for rates, and axis limits of graphs are created automatically by the application. *WAERS* does not allow a comparison of two or more RS curves since it does not compute RS for two or more groups at the same time. *RiskDiff* does not allow the assessment of statistical significance for the percent changes in risk and demographics. Future work will incorporate the integration of these features within these applications, although research in statistical methods must be developed specifically for *RiskDiff*.

## Conclusions

Unlike other similar applications, *REGSTATTOOLS* does not require local software installation and it is simple to use, fast and easy to interpret. *REGSTATTOOLS* is a set of web-based statistical tools intended for automated calculation of population indicators that any professional in the health or social sciences may require.

## Availability and requirements

**Project name:** REGSTATTOOLS.

**Project home page:** Access to the set of applications *SART*, *RiskDiff* and *WAERS* can be found through http://regstattools.net/.

**Operating system:** Platform independent for accessing the public web server.

**Programming language:** R and PHP.

**Requirements:** R statistical software available at http://www.r-project.org/ website is required for the functions implemented.

**License:** None.

**Any restriction to use by non-academics:** None.

## Abbreviations

ASR: Age standardized rate; CR: Crude rate; TR: Truncated rate; cumR: Cumulative rate; EAPC: Estimated annual percent of change; OS: Observed survival; RS: Relative survival; SIMR: Standardized incidence or mortality ratio; AF: Additional file; 95%CI: 95% confidence interval; RiskDif: A web tool for the analysis of the difference due to risk and demographic factors for incidence or mortality data; SART: Statistical analysis of rates and trends; WAERS: Web-assisted estimation of relative survival.

## Competing interests

The authors declare that they have no competing interests.

## Authors’ contributions

LE, RC, JG, LP and JR initially conceived the tool and were involved in its design. LE, RC and LP did the statistical analysis and the implementation in R JG implemented the web interface. JG and AI contributed to the collection, processing and interpretation of the data. All authors have been involved in drafting the manuscript and revising it critically. All authors approved the final version.

## Pre-publication history

The pre-publication history for this paper can be accessed here:

http://www.biomedcentral.com/1471-2458/13/201/prepub

## Supplementary Material

Additional file 1 freeware statistical tools for the analysis of disease population databases used in health and social studies”. Click here for file
