# Supplementary material for: REGSTATTOOLS: freeware statistical tools for the analysis of disease population databases used in health and social studies
Source: BMC Public Health. 2013 Mar 7;13:201. doi: 10.1186/1471-2458-13-201 (PMC3653818; doi:10.1186/1471-2458-13-201)
Supplement: Additional file 1 — freeware statistical tools for the analysis of disease population databases used in health and social studies”. [file 1471-2458-13-201-S1.doc]

**Additional File:**

**REGSTATTOOLS: freeware statistical tools for the analysis of disease population databases used in health and social studies.**

Laura Esteban^1^, Ramon Clèries^1,2^, Jordi Gálvez^1^, Laura Pareja^1^, Josep Maria Escribà^1^, Xavier Sanz^1^, Ángel Izquierdo^3^, Jaume Galcerán^4^, Josepa Ribes^1,2^

1. Cancer Registry of Catalonia - Plan for Oncology of the Catalan Government. IDIBELL, Hospital Duran i Reynals. Av. Gran Via de l’Hospitalet, 199-203, 08908 – L’Hospitalet de Llobregat , Catalonia-Spain

2. Department of Clinical Sciences, University of Barcelona, Barcelona 08907, Spain

3. Epidemiology Unit and Cancer Registry of Girona - Institut Català d'Oncologia (ICO). 17004, Girona, Catalonia - Spain

4. Tarragona Cancer Registry. Foundation Society for Cancer research and Prevention, Reus. Pere Virgili Health Research Institute, Spain

**Corresponding author:**

Ramon Clèries, PhD

Cancer Registry of Catalonia - Plan for Oncology of the Catalan Government. IDIBELL, Hospital Duran i Reynals. Av. Gran Via de l’Hospitalet, 199-203

08908 – L’Hospitalet de Llobregat , Catalonia-Spain

Tel.:+34 932607417

Fax: +34932607316

e-mail: [r.cleries@iconcologia.net](mailto:r.cleries@iconcologia.net)

**KEY WORDS:** web-application; prediction; standardized incidence mortality ratio; annual percent change; net percent change of rates; relative survival.

**ABBREVIATIONS FOR STATISTICAL TERMS:**

**ASR:** Age Standardized Rate; **CR:** Crude Rate; **TR:** Truncated Rate; **cumR:** Cumulative Rate; **EAPC:** Estimated Annual Percent of Change; **OS:** Observed Survival; **RS:** Relative Survival; **SIMR:** Standardized Incidence or mortality Ratio; **SM:** Supplement Material; **95%CI:** 95% Confidence Interval.

**Web-APPLICATIONS ABBREVIATIONS:**

**RiskDiff:** a web tool for the analysis of the difference due to risk and demographic factors for incidence or mortality data; **SART:** Statistical Analysis of Rates and Trends; **WAERS:** Web-Assisted Estimation of Relative Survival.

**Mathematical details of section “*Assessment of differences due to risk and demographic factors when comparing disease rates of two populations”***

To partition the differences between those due to differences in population demographics (size and structure) and those due to the differences in the risks we compare the rates in the two groups to an intermediate rate.

First, let us assume that we have $C_{1}$ and $C_{2}$ cases/deaths and a total population of $P_{1}$ and $P_{2}$and groups 1 and 2, respectively. The relative difference between $C_{1}$ and $C_{2}$ can be expressed as:

$\frac{C_{2}-C_{1}}{C_{1}}=\frac{C_{2}}{C_{1}}-1$ (1).

Let’s define the rates $S_{2}=\frac{C_{2}}{P_{2}}$ and $S_{1}=\frac{C_{1}}{P_{1}}$, equation (1) can be split into

$\frac{C_{2}}{C_{1}}-1=\frac{S_{2}P_{2}}{S_{1}P_{1}}-1=\left(\frac{S_{2}}{S_{1}}-1\right)+\left(\frac{P_{2}}{P_{1}}-1\right)\frac{S_{2}}{S_{1}}=g_{1}(C_{1},C_{2},P_{1},P_{2})+g_{2}\left(C_{1},C_{2},P_{1},P_{2}\right)$ (2).

Note that $g_{2}\left(C_{1},C_{2},P_{1},P_{2}\right)$ shows that change in the population size may lead to a change in the number of cases, which depends on the changes in the crude rate. The quantity $g_{1}\left(C_{1},C_{2},P_{1},P_{2}\right)$ is divided into one component due to the differences in risk and another component due to the differences in the population structure (age distribution).

Let us define $\lambda_{ik}$the rate in the i^th^ age group and in k^th^ strata (k = {1, 2}), and $w_{ik}=\frac{P_{ik}}{P_{.k}}$the proportion of population in the i^th^ age group and in k^th^ strata. We are interested in analyzing the differences in the crude rate between strata. Let us define $S_{3}=\sum_{i} \lambda_{i1}w_{i2}$, that is the rate in strata 1 applied to the population proportion in strata 2. Therefore

$\frac{S_{2}-S_{1}}{S_{1}}=\frac{\sum_{i} \left(\lambda_{i2}w_{i2}-\lambda_{i1}w_{i1}\right)}{\sum_{i} \lambda_{i1}w_{i1}}=\frac{\sum_{i} w_{i2}\left(\lambda_{i2}-\lambda_{i1}\right)}{\sum_{i} \lambda_{i1}w_{i1}}+\frac{\sum_{i} \lambda_{i1}\left(w_{i2}-w_{i1}\right)}{\sum_{i} \lambda_{i1}w_{i1}}$ (3),

where the first component on the right-hand side represents the proportion for the difference in the crude rate between strata 1 and 2 due to differences in the population structure and the second component represents the proportion of the differences due to risk.

**Table AFT1.** Age-groups file.


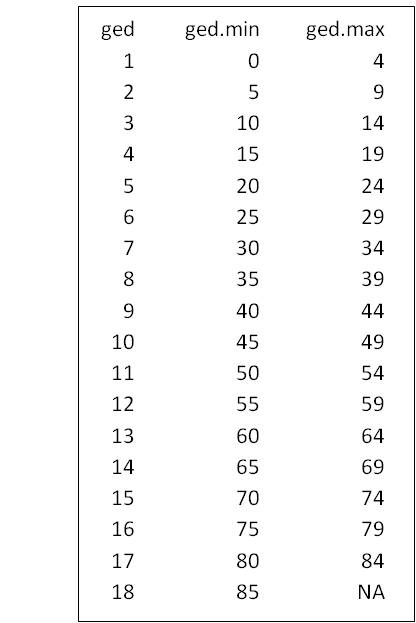


**Table AFT2.** World standard population's file.


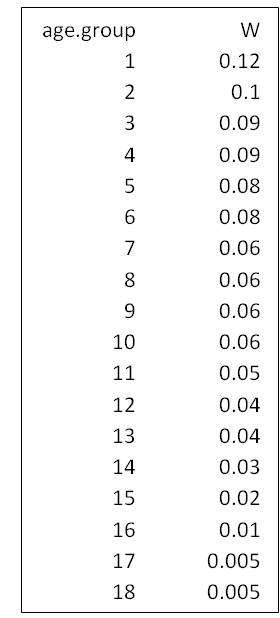


**Table AFT3.** Girona and Tarragona population distribution.


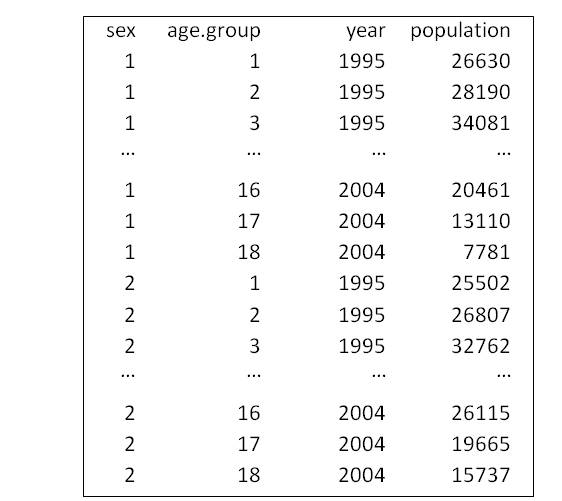


**Table AFT4.** Girona and Tarragona Cancer incidence ~~descriptive~~. 1995-2004.

|  | **Cancer site** | **Men** | | | | |  | **Women** | | | | |  |
| --- | --- | --- | --- | --- | --- | --- | --- | --- | --- | --- | --- | --- | --- |
|  |  | **N** | **CR** | **ASR(W)** | **TR(W)** | **CumR** |  | **N** | **CR** | **ASR(W)** | **TR(W)** | **CumR** |  |
|  | Kidney | 789 | 13.82 | 8.26 | 14.07 | 0.98 |  | 398 | 6.9 | 3.48 | 5.22 | 0.39 |  |
|  | Larynx | 901 | 15.78 | 10.4 | 22.77 | 1.26 |  | 29 | 0.5 | 0.34 | 0.84 | 0.04 |  |
|  | Lung | 4766 | 83.46 | 48.27 | 79.45 | 6.00 |  | 552 | 9.56 | 5.12 | 10.08 | 0.58 |  |
|  | Oesophagus | 479 | 8.39 | 5.32 | 11.03 | 0.64 |  | 67 | 1.16 | 0.55 | 0.97 | 0.06 |  |
|  | Oral cavity and pharynx | 1471 | 25.76 | 17.09 | 38.37 | 1.98 |  | 284 | 4.92 | 2.54 | 4.7 | 0.27 |  |
|  | Pancreas | 646 | 11.31 | 6.26 | 9.52 | 0.71 |  | 592 | 10.26 | 4.24 | 5.41 | 0.47 |  |
|  | Stomach | 1245 | 21.8 | 11.86 | 17.31 | 1.39 |  | 773 | 13.39 | 5.57 | 7.31 | 0.58 |  |
|  |  |  |  |  |  |  |  |  |  |  |  |  |  |

**N:** Number of cases , CR: Crude Rate x 100000 person-years; **ASR(W):** Age Standardized Rate x 100000 person-years to the World Standard Population, **TR(W):** Truncated Rate x 100000 person-years by the 35-64 age group to the World Standard Population; **CumR:** Cumulative Rate (percent) by the 0-74 age group

**Table AFT5.** Estimated Annual Percent Change and its 95 % Confidence Interval in Cancer incidence in Girona and Tarragona during 1995-2004.

|  | **Cancer Site** | **Men** | | |  | **Women** | | |  |
| --- | --- | --- | --- | --- | --- | --- | --- | --- | --- |
|  |  | **EAPC** | **LCI** | **UCI** |  | **EAPC** | **LCI** | **UCI** |  |
|  | Kidney | 0.28 | -1.51 | 2.1 |  | 2.45 | -1.69 | 6.76 |  |
|  | Larynx | -1.61 | -3.59 | 0.42 |  | 4.6 | -6.75 | 17.33 |  |
|  | Lung | -0.24 | -1.54 | 1.08 |  | 6.16 | 1.81 | 10.7 |  |
|  | Oesophagus | -2.45 | -5.24 | 0.41 |  | 2.89 | -2.85 | 8.97 |  |
|  | Oral cavity and pharynx | -3.23 | -5.43 | -0.97 |  | 3.52 | 0.91 | 6.19 |  |
|  | Pancreas | 2.77 | 0.73 | 4.85 |  | -0.34 | -3.05 | 2.43 |  |
|  | Stomach | -4.14 | -6.79 | -1.42 |  | -1.74 | -3.78 | 0.34 |  |
|  |  |  |  |  |  |  |  |  |  |

**EAPC:** Estimated Annual Percent Change, LCI: Lower Confidence Interval, **UCI:** Upper Confidence Interval.

**Table AFT6.** 2000-2004 cancer incidence aggregated data from Tarragona and Girona (target period)*.

**
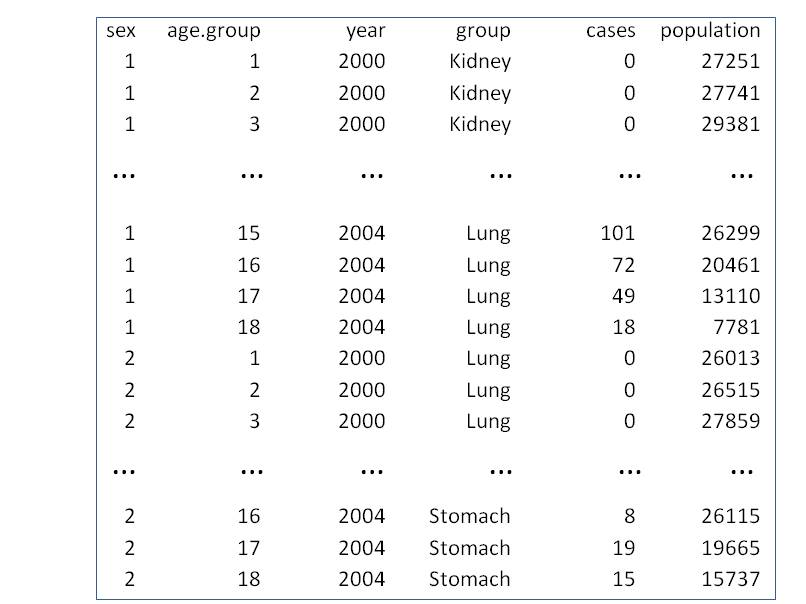
**

* It is the same structure for the reference period aggregated data.

**Table AFT7.** Standardized Incidence Ratio cancer incidence in Girona and Tarragona. 2000-2004 vs1995-1999.

|  | **Cancer Site** | **Men** | | |  | **Women** | | |  |
| --- | --- | --- | --- | --- | --- | --- | --- | --- | --- |
|  |  | **EAPC** | **LCI** | **UCI** |  | **EAPC** | **LCI** | **UCI** |  |
|  | Kidney | 0.28 | -1.51 | 2.1 |  | 2.45 | -1.69 | 6.76 |  |
|  | Larynx | -1.61 | -3.59 | 0.42 |  | 4.6 | -6.75 | 17.33 |  |
|  | Lung | -0.24 | -1.54 | 1.08 |  | 6.16 | 1.81 | 10.7 |  |
|  | Oesophagus | -2.45 | -5.24 | 0.41 |  | 2.89 | -2.85 | 8.97 |  |
|  | Oral cavity and pharynx | -3.23 | -5.43 | -0.97 |  | 3.52 | 0.91 | 6.19 |  |
|  | Pancreas | 2.77 | 0.73 | 4.85 |  | -0.34 | -3.05 | 2.43 |  |
|  | Stomach | -4.14 | -6.79 | -1.42 |  | -1.74 | -3.78 | 0.34 |  |
|  |  |  |  |  |  |  |  |  |  |

**SIMR:** Standardized Incidence or Mortality Ratio, **LCI:** Lower Confidence Interval, **UCI:** Upper Confidence Interval

**Table AFT8.** Aggregated data selection file for men*. 1995-2004.


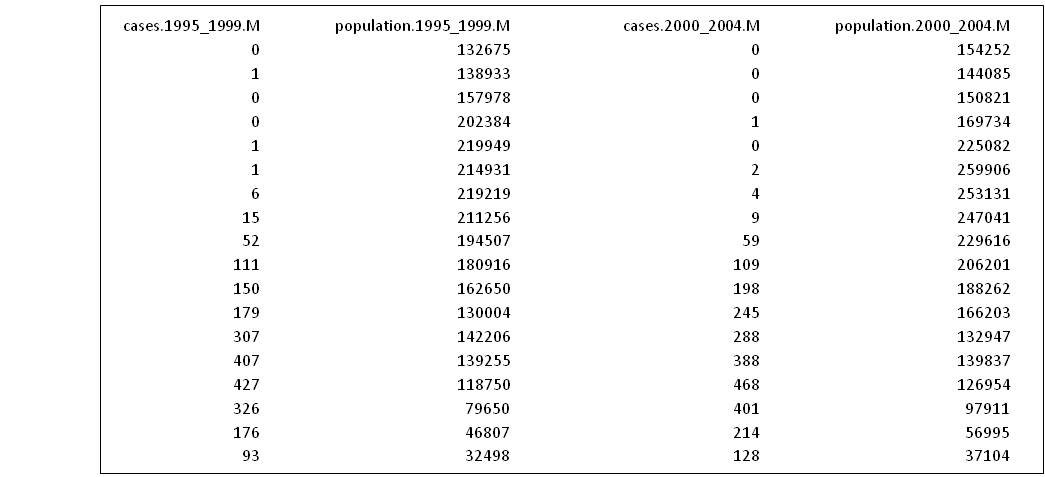


* The format file is the same for women.

**Table AFT9.** RiskDiff results for lung cancer incidence in men and women. 2000-2004 vs 1995-1999.

|  |  |  |  |  | |  |  |  |  |  | |  |  |  |
| --- | --- | --- | --- | --- | --- | --- | --- | --- | --- | --- | --- | --- | --- | --- |
|  | **Change in** |  | **Men** | | | | |  | **Women** | | | | |  |
|  |  |  | **Crude Rate** | | **%** | **Number** | **%** |  | **Crude Rate** | | **%** | **Number** | **%** |  |
|  | Risk |  | 0.75 | | 0.91 | 20.49 | 0.91 |  | 1.52 | | 17.53 | 42.07 | 17.53 |  |
|  | Structure |  | 0.78 | | 0.95 | 21.34 | 0.95 |  | 0.24 | | 2.72 | 6.52 | 2.72 |  |
|  | Size |  | NA | | NA | 220.17 | 9.78 |  | NA | | NA | 23.42 | 9.76 |  |
|  | Net Change |  | 1.54 | | 1.86 | 262.01 | 11.63 |  | 1.75 | | 20.24 | 72.00 | 30.00 |  |
|  |  |  |  | |  |  |  |  |  | |  |  |  |  |

**Table AFT10.** Individual records file* to WAERS analysis. Patients with lung cancer in Tarragona and Girona during the period 2000-2004 in men.


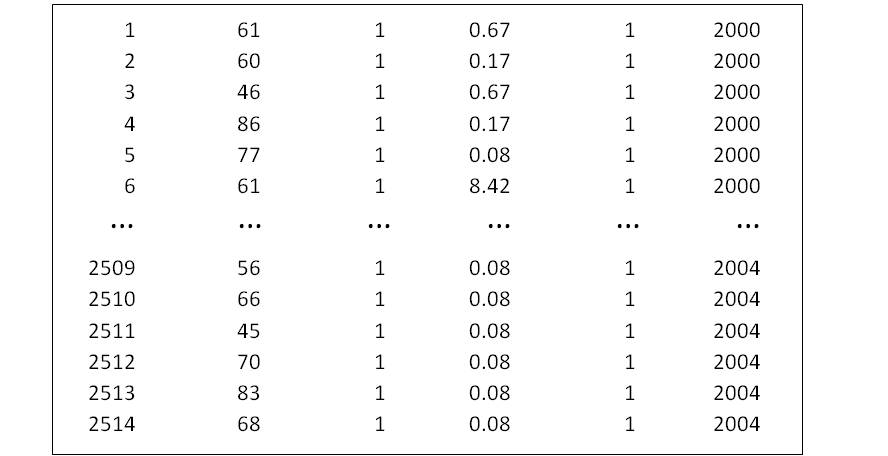


1st column: patient ID ; 2nd column: age at the diagnostic; 3rd column: sex; 4th column: follow-up ; 5th column: status, 6th column:year of the last follow-up.

* The format file is the same for women and for other period.

**Table AFT11.** Catalonia population projections by age group and sex in 2014 (IDESCAT).


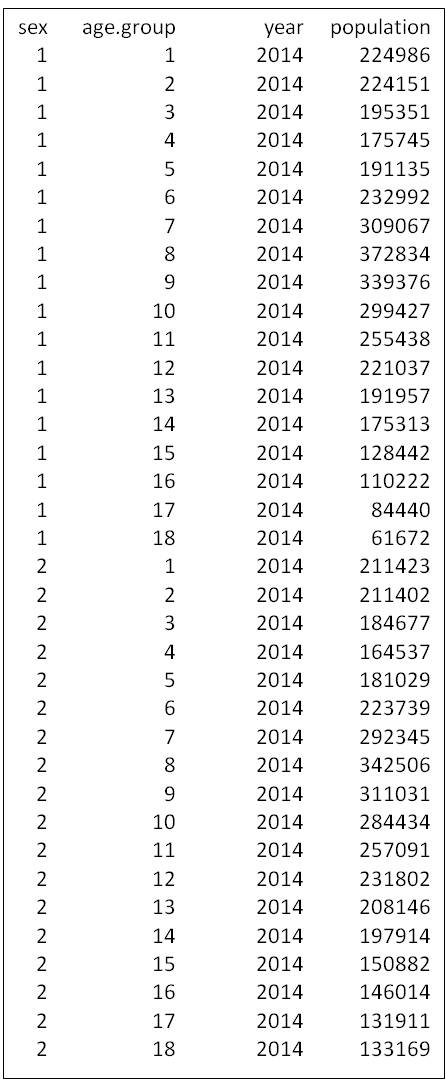


**Table AFT12.** Model selection by group and sex based on the1995-2004 period from Girona and Tarragona data.


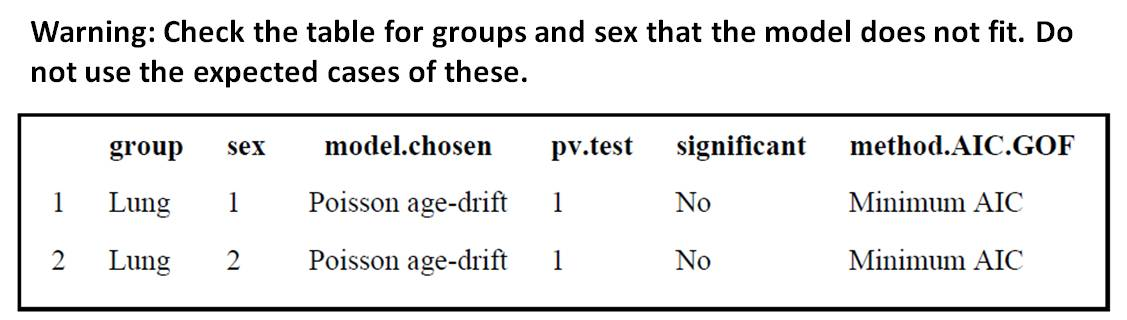
**group:** Group of interest; **sex:** 0= Both sexes, 1=Men, 2= Women; **model.chosen:** The fitted model chosen to predict; **pv.test:** The p-value of the model goodness of fit test; **significatn:** Is the precedint p.value significant?; **method.AIC.GOF:** The method used for model selection (AIC: Akaike Information Criterion; GOF: chi-square goodness of fit test).

## Table AFT13. Catalonia lung cancer ~~incidents~~ incidence projections 2014 ~~descriptive~~.

|  |  |  |  |  |  |  |  |
| --- | --- | --- | --- | --- | --- | --- | --- |
|  | **Sex** | **N** | **CR** | **ASR(W)** | **TR(W)** | **CumR** |  |
|  | Men | 3085 | 81.32 | 46.82 | 76.37 | 5.77 |  |
|  | Women | 777 | 20.11 | 10.74 | 19.64 | 1.16 |  |
|  |  |  |  |  |  |  |  |

N: Number of cases , CR: Crude Rate x 100000 person-years, ASR(W): Age Standardized Rate x 100000 person-years to the World Standard Population, TR(W): Truncated Rate x 100000 person-years by the 35-64 age group to the World Standard Population, CumR: Cumulative Rate (percent) by the 0-74 age group

**Table AFT14.** Lung expected cases in Catalonia by sex in 2014.


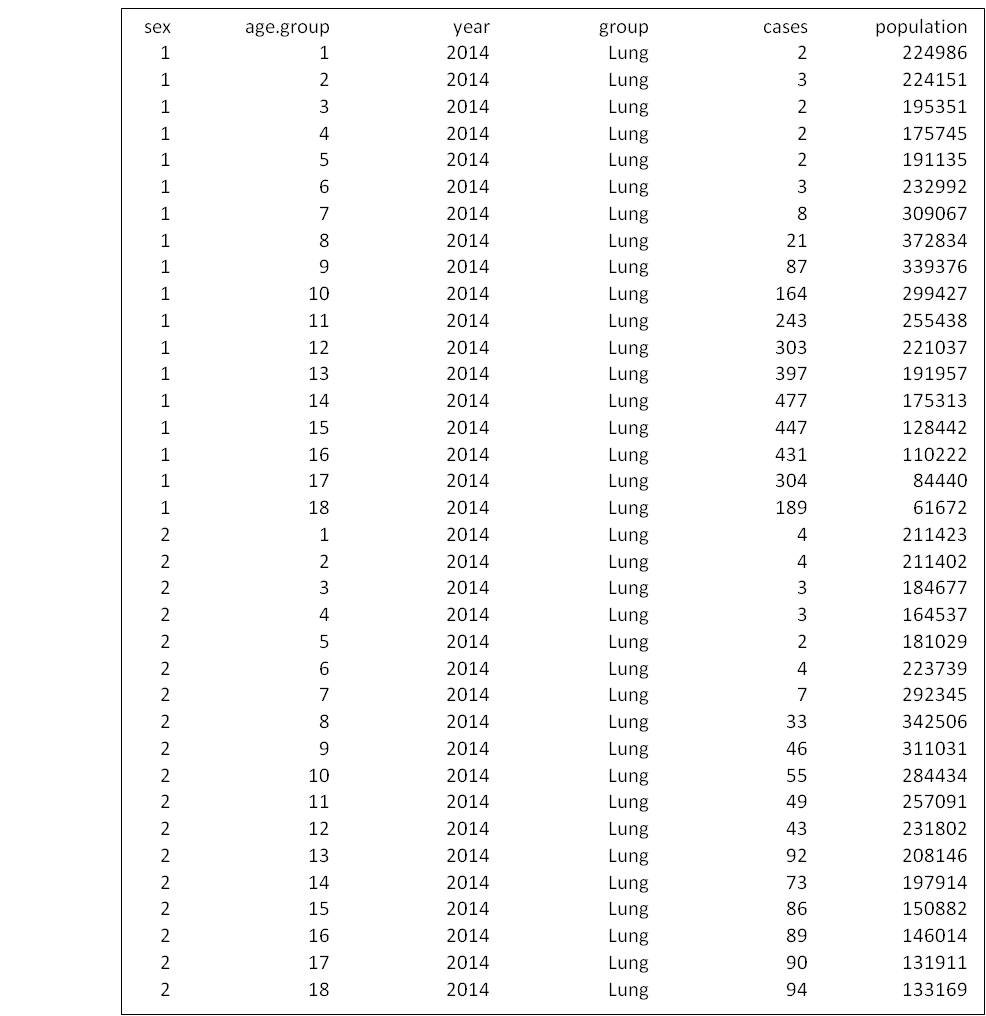


**Table AFT15.** Percentage use of each one of the applications included in REGSTATTOOLS.

|  |  |  |  |  |  |  |
| --- | --- | --- | --- | --- | --- | --- |
|  | **Application** | **Period*** | **Type of user**** | | | |
|  |  |  | **Cancer** | **Cancer** | **Other** |  |
|  |  |  | **Registries** | **Control** |  |  |
|  | **SART** | *June 2011 to November 2012* | 65.94 | 31.89 | 2.17 |  |
|  | **WAERS** | *June 2005 to November 2012* | 60.71 | 13.13 | 26.16 |  |
|  | **RiskDiff** | *June 2009 to November 2012* | 35.71 | 21.43 | 42.86 |  |
|  |  |  |  |  |  |  |

*Period of time considered for each application. All applications were available a little earlier than the beginning of the period considered.

**The type of users considered are Cancer Registries, Cancer Control Units and Other. Cancer Control Units consider national agencies of data collection in each country such as national institutes of statistics of health not focused only on cancer. Other include Universities and research centers.

**Figure AFF1.** Age standardized incidence rate* ranking of tobacco-related tumors, 1995-2004.

Men

Women


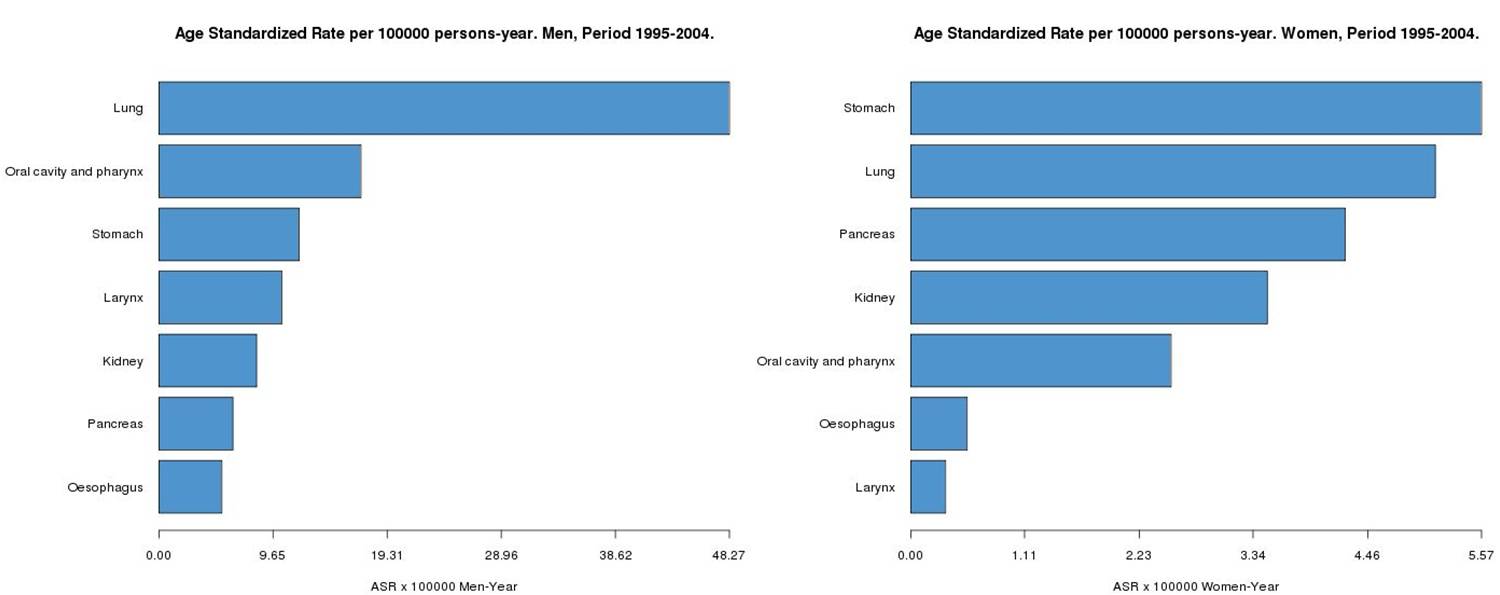


*~~This figure it could be also obtained for the number of cases, crude rate, truncate rate or cumulative rate in the Descriptive application.~~ Figures like these could be also obtained for the number of cases and crude truncate or cumulative rates using the Descriptive application.
